# Identification of the Components of a Complex Intervention to Develop Clinical Leadership in Specialist Clinical Nurses: A Qualitative Study with Interviews and Stakeholders’ Consensus

**DOI:** 10.2147/JHL.S581117

**Published:** 2026-05-21

**Authors:** Antonio Palermo, Monica Bianchi, Shaila Cavatorti, Loris Bonetti

**Affiliations:** 1 Nursing Department Direction, Ospedale Regionale Bellinzona e Valli, Ente Ospedaliero Cantonale, Bellinzona, Ticino Canton, Switzerland; 2 Department of Business Economics, Health and Social Care, University of Applied Sciences and Arts of Southern Switzerland (SUPSI), Manno, Ticino Canton, Switzerland

**Keywords:** shared governance, leadership, specialist nurse, complex intervention, MRC framework, qualitative research

## Abstract

**Purpose:**

Healthcare systems have undergone changes due to ageing populations and responding to new care needs, with developments in nursing in terms of organization, skills and new roles. The introduction of specialist clinical nurses (SCN) in all departments was considered crucial to expand the professional development possibilities for nurses. The purpose of this study was to examine SCNs perceptions of their role and clinical leadership, and to identify the components of a complex intervention aimed to improve clinical leadership.

**Methods:**

Qualitative research was performed in a multisite Hospital, via semi-structured interviews with SCNs, involved with a purposive sample, to investigate perceptions of their role and barriers/facilitators to clinical leadership and its development. A review of relevant literature informed the interview guide and a thematic analysis of data was performed. The results and literature relating to clinical leadership were synthesised for the first phase, of the Medical Research Council framework (development of the intervention) to develop a complex intervention focusing on clinical leadership. A consensus meeting was performed with SCNs and nurse managers to define the intervention.

**Results:**

Seven interviews were conducted with SCNs. Five macro themes were identified contributing to clinical leadership as a SCN: personal characteristics and competencies, collaboration and relationships, organization and culture of teams, leadership in the role and patient. During the consensus meeting the results were discussed and participants highlighted existing resources for leadership and those requiring development. A complex intervention to improve clinical leadership was defined, consisting of an introductory pathway, basic training package, coaching/support in practice and group work.

**Conclusion:**

The study has identified a variety of factors that could influence clinical leadership and how they could be applied within this clinical role. Elements to further develop clinical leadership for SCNs have been outlined taking into account the local context and the need to introduce additional interventions based on the needs of the individual and the team.

## Introduction

Clinical leadership is a relatively new concept in healthcare, but it is considered crucial for influencing practices, innovation, change and improvement in care.^1^ Clinical leadership is defined as the process of influencing point-of-care innovation and improvement in organizational processes and care practices to achieve quality and safety of care outcomes.^2^ It differs from managerial leadership in the additional requirement technical competency within the area of practice.^3^ Effective clinical leadership may have positive impact on the work environment, enhancing nurses’ professional growth and job satisfaction.^4^

Clinical skills, effective communication and the ability to positively influence teams are key attributes of clinical leadership. Leadership development should involve all nurses, regardless of their role, through training, mentoring, and organizational support. Promoting a culture of feedback and practical development opportunities can strengthen clinical leadership, improving patient care and the work environment.^5^,^6^

Unlike leadership in general, clinical leaders operate at high levels of clinical interaction, without necessarily holding managerial positions. Clinical competence is fundamental to clinical leadership because of its impact at point-of-care. According to Stanley and Stanley,^1^ competence acquired through experience and credibility among peers are key attributes. Nurses with clinical leadership are effective collaborators and communications, capable of coordinating patient care.^5^ Within the local context, clinical leadership is fundamental within the role of the Specialist Clinical Nurse (SCN). This figure promotes evidence-based nursing practice, along with knowledge and skill development of the nursing team in order to guarantee high quality care.

It is expected SCNs within this institutionally recognised role, are able to demonstrate their clinical leadership through various activities including coordination of complex patient care situations, intra- and inter-professional collaboration and facilitation of change within the team.

Pizzirani^7^ highlighted gaps in the evaluation of the effectiveness of clinical leadership development programs. Despite the priority given to these programs, there is little evidence of the impact clinical leadership has on patient outcomes. Other studies show that clinical leadership can improve the quality of care through motivation, coaching and training.^8^ Clinical leadership is considered crucial for addressing issues such as complexity, change, safety and quality of care^2^ and is essential for a positive work environment and professional development.^9^ It can be considered a shared responsibility, exercised by every healthcare professional regardless of their position in the healthcare system.^10^

Leadership training can improve the hospital work environment by promoting team competence and decision-making autonomy.^11^

Although nurses believe that formal qualifications are necessary to develop leadership skills, leadership activities at the patient’s bedside are an integral part of nursing practice.^9^ Nurses with greater clinical experience are often considered informal leaders, able to influence their colleagues even without holding a formal role.^10^

In light of this, developing clinical leadership is essential to improving the quality of care.

Nevertheless, to our knowledge, few studies have examined what strategies could be implemented to promote the development of clinical leadership among healthcare professionals. Also, within the local context, no structured pathway for the development of clinical leadership was available for SCNs and particular to this role, and it is based on this knowledge gap that we decided to develop this study. As it is suggested that nurse leaders should reflect on and take responsibility for development of their own leadership expertise^12^ it was felt appropriate to involve SCNs in this process.

Therefore, the purpose of this study was to examine SCNs perceptions of their role and clinical leadership, and to identify the components of a complex intervention aimed to improve clinical leadership.

The specific objectives defined for the study were:

- To investigate the perceptions of specialist clinical nurses (SCN) in terms of their role and areas of clinical leadership.
- To investigate the presence of clinical leadership improvement interventions in the daily clinical practice of SCN.
- To identify the elements and behaviors that foster clinical leadership development in SCNs.
- To identify the main components of a complex intervention to be implemented to promote clinical leadership development using the Medical Research Council (MRC) Framework.

## Materials and Methods

### Study Design

The project used a generic qualitative research method.^13^,^14^ Unlike specialized frameworks such as ethnography or grounded theory, generic qualitative research adopts a non-categorical approach. Its primary objective is to provide a comprehensive description of a phenomenon without the constraints of a specific theoretical tradition.^13^ The study was conducted from April 2022 to September 2023.

In order to identify the elements and behaviours that facilitate clinical leadership development in Specialist Clinical Nurses (SCNs) and to identify the main components of a complex intervention to be co-constructed and implemented to promote clinical leadership development, the Medical Research Council (MRC) framework was used.

The MRC framework guides the development, evaluation and implementation of complex health interventions through four phases: intervention development or identification, feasibility, evaluation and implementation. As the development of clinical leadership is a complex, multi-factorial phenomenon involving multiple variables, the framework is pertinent in this setting. Each stage includes key elements that must be considered continuously, such as: context, reference theory - if any, involvement of professionals, identification of uncertainties, refinement of the intervention and economic considerations. These elements must be integrated and revised to effectively guide the process of creating the complex intervention.^15^

In order to define the elements underlying the co-construction of the complex intervention, a consensus meeting with experts was adopted.^16^,^17^

### Setting and Population

The study setting was a public multi-specialty hospital in the Italian-speaking part of Switzerland. The population involved the study was composed of SCNs working in 16 departments out of a total of 17 within the nursing service. These professionals perform both direct and indirect patient care activities, such as supporting nursing teams (e.g., implementation of guidelines), nurse managers, and the hospital organization. Time dedicated to these activities varies between 10% and 50% of her total monthly working hours.

### Sampling

#### Interviews

To identify participants for the semi-structured interviews, purposeful sampling was adopted according to reasoned choice, recruiting participants who were experts in the phenomenon of interest. Data saturation was reached when no new relevant information emerged.^18^,^19^

The sample for the consensus meeting was larger and heterogeneous with purposeful sampling according to reasoned choice.^18^,^19^

#### Consensus Meeting

In the consensus meeting, professional figures were chosen who could give information about the peculiarities of clinical leadership from different perspectives (clinical, management and research) and who knew the local context. This choice was made in order to create a complex intervention starting with the professionals and taking into account the local reality. For this reason, both SCNs, managers and research experts were involved. Participants were contacted individually by the first author, who organized the consensus meeting.

### Data Collection Methods

#### Interviews

**Figure 1:**
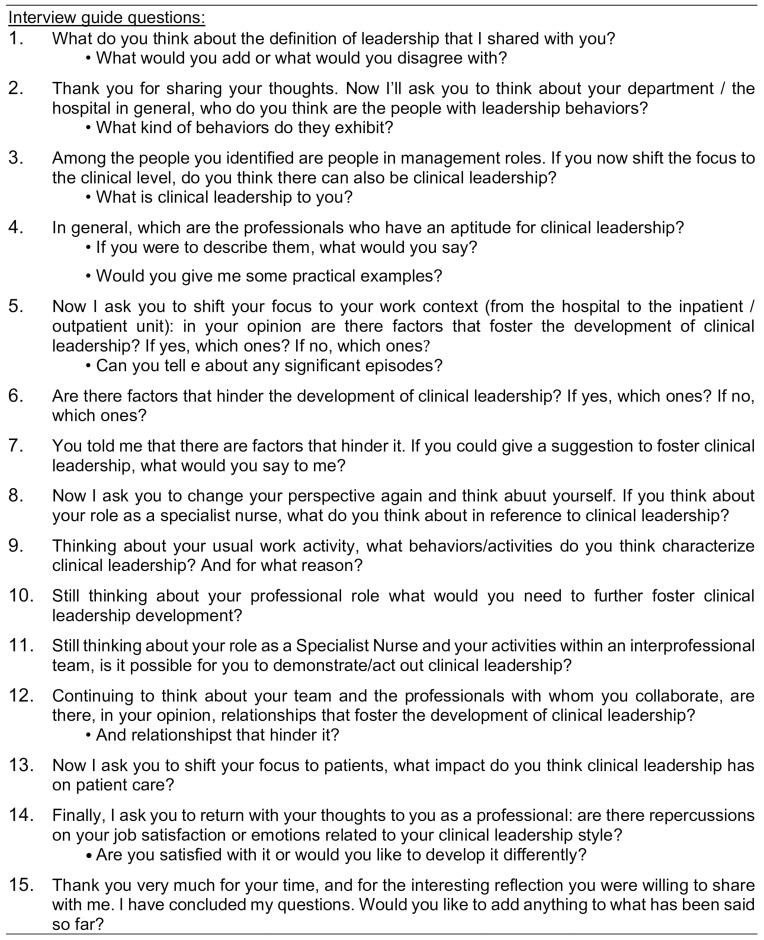
Questions for semi-structured interviews.

Data was collected through semi-structured individual interviews with Specialist Clinical Nurses following the guiding questions shown in Figure 1.

All interviews were conducted by the first author, in a quiet environment free from distraction. Interviews were digitally recorded and transcribed. Before data collection started, a pilot interview to test the interview guide was conducted.

#### Consensus Meeting

Subsequently, considering the data collected from the interviews, and to define and reach a consensus on the constituent elements of a complex intervention aimed at developing clinical leadership, a consensus meeting was held involving SCNs and hospital managers. The consensus meeting was organized considering models proposed by Arakawa and Bader^16^ and James and Warren-Forward.^17^ It was prepared by the research team and conducted by the first author. All the participants were in presence in a dedicated room with chairs in semicircles.

The consensus meeting was held presenting the results of the field research derived from the thematic analysis of the interviews and evidence from the literature review conducted prior to the start of the study. During the consensus meeting, the information was discussed and compared in order to identify possible strategies for clinical leadership. Finally, the results obtained were compared with existing literature to define the specifics of the complex intervention. This comparison made it possible to outline future steps and ensure that the proposed intervention was in line with scientific evidence, the practical needs of the local context, and was a shared intervention and not imposed top-down. Interviews and the consensus meeting were audio-recorded and transcribed verbatim by a professional transcription service. All recordings were stored in a password protected secure folder, accessible exclusively to the research team.

### Data Analysis

Transcripts were analyzed using the thematic analysis proposed by Braun and Clarke,^20^ which identified the main recurring and relevant themes, with an inductive approach. Following the initial interviews analysis by the first author, a formal codebook was established to guide the thematic development. These codes were refined through discussions with the senior author and received final approval from the full research team. Subsequently, the consensus meeting method^16^ was applied to validate these results and define the key elements of the complex intervention.

### Trustworthiness and Rigor

In the study, Guba and Lincoln’s^21^ criteria were followed to ensure methodological rigor, focusing on credibility, transferability, dependability and confirmability.

- Credibility: A reflexive approach was taken, using bracketing to avoid personal influences.^22^ The interviews were transcribed, reviewed, and cross-checked against the audio recordings multiple times. Data analysis was supported by NVivo 12©, which facilitated the development of codes, the definition of themes, and the systematic organization and extraction of quotes for the study’s findings. The process was supervised by two qualitative research experts.
- Trasferability: A detailed description of the procedures and social-health context was provided, allowing other researchers to assess the applicability of the results in similar settings.
- Dependability: The thematic analysis was conducted individually and then shared and validated by a second experienced researcher. The results were reviewed by a researcher experienced in qualitative methodology for confirmation.
- Confirmability: In the report, excerpts from the interviews were used with the exact words of the participants. The consensus meeting produced minutes, reviewed by all participants to ensure the accuracy of the results.
- Traceability: An updated action plan was maintained that narratively described the experiences and decisions made, allowing the project to be reconstructed at any time.

These provisions ensured the rigor, validity and reliability of the study results.

To mitigate potential hierarchy and social desirability biases, all participants were guaranteed strict anonymity and informed that their individual responses would not be shared with their organization or supervisors, thus fostering a safe environment for open disclosure. Moreover, the interviews were conducted by the first author, who adopted a neutral, non-judgmental stance and utilized open-ended, non-leading questions. This approach encouraged participants to share authentic experiences rather than socially desirable answers. Finally, the researchers engaged in constant reflexivity throughout the coding process, discussing potential personal biases during regular debriefing sessions to ensure that the final themes remained grounded in the participants’ actual perspectives.

### Ethical Considerations

The study was conducted in accordance with the Declaration of Helsinki.^23^

According to Swiss legislation, the study did not require Ethics Committee approval because it was aimed at professionals employed in our region. Data were anonymized to ensure confidentiality and privacy by creating an alphanumeric file that allowed only the researcher to know participant identity. Individuals in both the interviews and the consensus meeting provided written informed consent prior to participation. The participants informed consent included publication of anonymized responses/direct quotes.

## Results

### Interviews

Semi-structured interviews were conducted with SCNs representing all clinical areas of the hospital. All seven SCNs invited to the study provided informed consent to participate. The majority were female, employed full-time, with 3 to 6 years of professional experience (Table 1). Table 1 details also the participants of the consensus meeting, which included nine SCNs, one manager, and one researcher.

**Table 1:** Participants Characteristics

Participant Characteristics	Interviews (n=7)	Consensus Meeting (n=11)	Total (N=18)
**Sex**	**n (%)**	**n (%)**	**n (%)**
- Male	1 (14.3)	2 (18.2)	3 (16.7)
- Female	6 (85.7)	9 (81.8)	15 (83.3)
**Age, n (%)**	**n (%)**	**n (%)**	**n (%)**
- 20–35	4 (57.1)	5 (45.5)	9 (50.0)
- 36–50	3 (42.9)	6 (54.5)	9 (50.0)
**Diploma in Advanced Studies**	**n (%)**	**n (%)**	**n (%)**
- Yes	5 (71.4)	7 (63.6)	12 (66.7)
- No	2 (28.6)	4 (36.4)	6 (33.3)
**Other academic course**	**n (%)**	**n (%)**	**n (%)**
- Yes	6 (85.7)	9 (81.8)	15 (83.3)
- No	1 (14.3)	2 (18.2)	3 (16.7)
**Total Work experience (years)**	**n (%)**	**n (%)**	**n (%)**
- 2–5	1 (14.3)	2 (18.2)	3 (16.7)
- 6–15	5 (71.4)	3 (27.3)	8 (44.4)
- 16–25	1 (14.3)	6 (54.5)	7 (38.9)
**Employment status (%)**	**n (%)**	**n (%)**	**n (%)**
- 70–80%	2 (28.6)	0 (0.0)	2 (11.1)
- 90–100%	5 (71.4)	11 (100)	16 (88.9)
**Work area**	**n (%)**	**n (%)**	**n (%)**
- Surgery	2 (28.6)	2 (18.2)	4 (22.2)
- Medicine	3 (42.9)	3 (27.3)	6 (33.3)
- Critical care	1 (14.3)	3 (27.3)	4 (22.2)
- Outpatient	1 (14.3)	1 (9.1)	2 (11.1)
- Research	0 (0.0)	1 (9.1)	1 (5.6)
- Management	0 (0.0)	1 (9.1)	1 (5.6)
**Experience as SCN (years)**	**n (%)**	**n (%)**	**n (%)**
- 1–2	2 (28.6)	4 (44.4)	6 (37.5)
- 3–6	4 (57.1)	4 (44.4)	8 (50.0)
- 7–10	1 (14.3)	1 (11.2)	2 (12.5)
	/	2 NA*	2 NA*

**Note**: *NA- Not applicable (Manager/Researcher).

The duration of the interviews ranged from 30 to 52 minutes. From the thematic analysis, 20 themes were identified and grouped into 5 macro-themes (Table 2).

**Table 2:** Thematic Map

Macrotheme	Theme	n. Documents*	n. Refs.**
Personal characteristics and competencies	Autonomy	7	24
	Expectations	7	49
	Knowledge	6	10
	Awareness and leadership behaviors	7	53
	Personal characteristics	7	41
Collaboration and Relationships	Collaboration with superiors	7	50
	Collaboration and discussion with colleagues	7	84
	Communication	6	23
	Support in change	6	13
Organization and culture of teams	Work environment	6	25
	Training	6	21
	Organization	7	30
	Empowerment	7	23
	Recognition	6	18
	Time variable	7	17
Leadership in the role	Role clarity	7	22
	Formal roles	7	18
	Informal roles	7	24
Patient	Patient wellbeing	6	10
	Quality and safety of care	6	13

**Notes**: *n. Documents: Number of interviews in which the theme emerged. **n. Refs.: Total number of references or mentions of the theme across all interviews.

The themes contained in the first macro-theme, “Personal characteristics and competencies,” explored personal characteristics and skills needed for leadership, highlighting autonomy in work, personal and organizational expectations, and the importance of knowledge and awareness. Participants emphasized that leadership begins with oneself, requiring awareness and personal self-criticism on communication skills.

[.] you start from factors that are however personal first of all, to reflect on what kind of leader you want to be. (Id5)

[…the leader] is a person who welcomes your experiences, makes you think, doesn’t take a firm stance [.] tries to mediate and from a certain point of view cares about you. (Id2)

The second macro-theme, “Collaboration and Relationships,” grouped together themes related to collaboration and relationships, highlighting the importance of collaborating effectively with superiors and colleagues for leadership development. It was emphasized that the relationship with the Ward Manager can positively or negatively influence SCN leadership, depending on the collaborative dynamic. Communication was seen as crucial to successful professional relationships, with a focus on transparency and building positive relationships.

Sharing with others could also help in acquiring behaviors. (Id4)

[…] I would like to improve myself and my leadership, to compare myself with people who have more experience than me […]. (Id3)

In the third macro-theme, “Organization and culture of teams,” the themes delved into hospital organization and team culture, highlighting how a good working climate and a growth-oriented culture are key to facilitating leadership. Training is seen by participants as essential to meeting the challenges of the role, a particular emphasis was placed on specific training in clinical leadership. Empowerment was seen as a way to increase the visibility and recognition of the SCN within the team.

[…] empowerment serves not only to promote good clinical practice but also a good working environment […]. (Id2)

The aspects that can still facilitate for me is a team that trusts the leader and makes all employees feel important and involved. (Id3)

Talking about it, thematizing the topic of clinical leadership could be a favorable factor in its development. (Id3)

The fourth macro-theme, “leadership in the role,” grouped themes exploring leadership in the specific role of the SCN, with a focus on role clarity and the distinction between formal and informal roles. Clear role definition was considered crucial for SCN autonomy and recognition, and leadership was also recognized by participants in informal roles, such as that of the ward nurse.

[…] it is important to define the role, to then have means to develop this leadership. (Id2)

[…] also comes through clarity of roles and mandates basically. (Id7)

Finally, the fifth macro-theme “patient” included themes that highlight how good clinical leadership can improve the well-being and quality of care provided.

[…] good clinical leadership allows a good return home and then being able to stay at home [.] comprehensive care; that is in the sense, there’s the patient however maybe there is also a particular family situation. So you also try to take charge of that situation, considering well-being towards the patient. (Id7)

Subsequently, a consensus meeting was held lasting 3 hours, which was a central step in outlining an intervention aimed at promoting clinical leadership of SCNs. Based on the results of a literature review distributed to participants and the results of the interview analysis, the group found consistency among the findings that emerged, although the literature did not always clearly define the role of the SCN in the specific context.

No divergent opinions were observed and a unanimous consensus was reached.

During the consensus meeting, a complex intervention was co-constructed with participants, the components of which include a structured professional development course, emphasizing clinical leadership training, communication, and peer support. Individual self-assessment was identified as key to personalizing the pathway, which is designed to be continuous and adaptable according to personal and organizational needs and goals.

The discussion underlined the need to prioritize interventions and target audiences, beginning with leadership self-assessment to guide detailed pathway definition.

**Figure 2:**
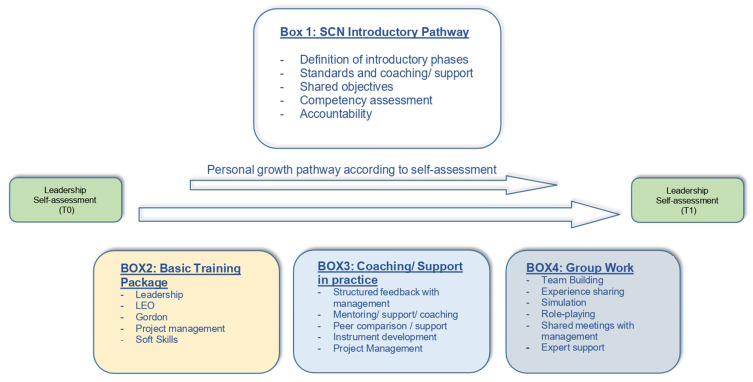
Elements of the complex intervention.

**Notes**: LEO= leading and empowered organization education; Gordon= communication skill development education; T0= Time 0 (Leadership Self-assessment before intervention implementation); T1= Time 1 (Leadership Self-assessment after intervention implementation).

In summary, the consensus meeting consolidated an integrated, theoretical-practical approach to NSC clinical leadership development, emphasizing collaboration, ongoing support, and personalization of the intervention in response to individual and organizational needs. Figure 2 shows the constituent elements of the complex intervention, derived from the consensus meeting.

Overall, a picture emerges in which the clinical leadership development of SCNs is strongly influenced by personal characteristics, professional relationships, and the organizational and cultural environment in which they operate.

## Discussion

The purpose of this study was to examine SCNs perceptions of their role and clinical leadership, and to identify the components of a complex intervention aimed to improve clinical leadership.

Analysis of the data from the semi-structured interviews and the results of the consensus meeting revealed multiple elements.

Semi-structured interviews highlighted that clinical leadership of SCNs was influenced by diverse interconnected factors including personal characteristics, professional competencies (autonomy, responsibility and reflection on practice), quality of relationships and collaboration with colleagues and superiors, organizational climate and team culture as well as role clarity.

The consensus meeting facilitated the definition of key elements within an intervention for leadership development, underlining the importance of a structured pathway shared with the organization, including specific training programs (communication, collaboration, empowerment) and instruments such as simulation and moments of peer discussion. The identification of the key elements of the complex intervention to develop clinical leadership demonstrated that current available leadership programs taken individually, responded only in part to the needs of SCNs. Together, these results contribute to clarify factors favouring clinical leadership of SCNs and the characteristics of an organizational intervention aimed to effectively sustain its development.

### Characteristics of the Person and Competencies

An initial consideration that emerged from this study concerns how personal qualities and skills acquired over the years are critical to the development of leadership of SCNs. A crucial building block is autonomy: it must be pursued in the work of SCNs and passed on to colleagues to positively influence nursing performance, their experience, and organizational outcomes.^24^,^25^ Clinical leadership is fostered if nurses can make autonomous decisions using their clinical skills and critical thinking abilities, demonstrating accountability.^24^,^25^

Another aspect that emerged concerns strategies to facilitate clinical leadership, they should simultaneously address knowledge and skills to solve practical problems collaboratively, with assumption of responsibility, competence and autonomy. This would promote high quality care, satisfaction and retention of nurses.^26^

For SCNs, the theme of autonomy is linked to the theme of trust, understood as the expectation placed on colleagues, superiors and the institution in general, which is considered crucial to the development of one’s role and leadership. When superiors express confidence in the ability of SCNs to deliver high-level performance, they feel important to the organization and more empowered to make decisions, be proactive, and demonstrate greater commitment to activities.

Respondents believe that conscious development of one’s leadership and performance comes through working on oneself to improve one’s characteristics, leveraging personal qualities such as positive attitude, reflective practice, and leadership for change, but also working on relationships with colleagues and superiors for peer learning. These thoughts are confirmed by Stanley and Stanley,^1^ who find that the central attributes of the clinical leader are based on clinical competence and credibility gained among peers.

The attributes of clinical leadership focus on the clinical, team and personal qualities needed to build healthy work environments and promote resilience among nursing staff. Respondents recognize the importance of being able to accommodate the experiences of others and mediate conflict within the team, requiring predisposition to listen and understanding of different perspectives, as expressed by several authors.^5^,^8^,^10^

### Collaboration and Relationships

The relationship between SCNs and their hierarchical superiors is fundamental to the development of clinical leadership. Participants agreed on the importance of effective collaboration and open and constructive discussions with their superiors, allowing them to create a network of relationships based on mutual trust and communication. This type of relationship is essential for the development of strong and enduring clinical leadership, allowing professionals to work in synergy and share their experiences and expertise, fostering continued professional growth.^27^

Some authors suggest that professional growth is fostered by a process of shared governance, understood as shared decision-making between nurses and nurse managers.^28–30^

In addition to collaboration with superiors, participants emphasize the importance of collaboration and discussion with colleagues (peer to peer) for the growth of their role and leadership. The benefits of working as a team on hospital projects, attending regular meetings to share common themes, and having reference people who serve as inspiration/example are mentioned.^31^

Participants believe that honest and transparent communication with colleagues and patients is essential, considering it a key element in clinical leadership development. Good communication leads to greater collaboration and supports clinical leadership, while poor communication can impede collaboration and negatively affect clinical leadership.^6^

### Organization and Culture of Teams

Participants agreed that a good working climate fosters leadership and that it is everyone’s responsibility to help create it. A positive and collaborative climate encourages sharing of projects, ideas and improves communication among team members. Conversely, a tense climate can hinder the achievement of goals and overburden staff. Transformational leadership is important in creating high quality and responsible work environments by promoting clinical leadership practices at the patient’s bedside to avoid adverse events.^5^,^12^

Participants believe that improving a work climate also means fostering a culture that views mistakes as opportunities for improvement and team growth rather than blame. This requires understanding the motivations behind others’ behaviors rather than judging them.^32^

As stated by RNAO^12^ leadership training is seen as an additional support to facilitate its development, along with networking and access to resources that promote the growth and involvement of professionals.

The evidence suggests that favourable working environments, access and opportunity for training and occupational health protection have a crucial role in reinforcing professional development and promoting sustainable leadership capacity among healthcare professionals.^33^,^34^

### Leadership in the Role

Interviews show that role clarity is critical for SCNs, as it allows them to be recognized and have autonomy in their work. Role definition and understanding the difference between hierarchical superior and leader, as well as between clinical leadership and management leadership, are seen as important for SCN leadership development.

Some participants point out that in recent years the role of the SCN has become more defined thanks to corporate culture, SCN coordination, and collaboration with other healthcare professionals. This has made the figure of the SCN more visible and allowed new skills and practices to be discovered.

Leadership is also recognized in people who do not have a formal hierarchical position as also confirmed by Mianda and Voce,^10^ in fact, they state that clinical leadership can be a shared responsibility carried out by any competent professional regardless of position within the health care system.^10^ These professionals are able to influence care outcomes and pursue bottom-up care development.^9^

Overall, participants believe that leadership is an important characteristic for the role of the SCN in both formal and informal settings.

### Discussion Results of the Consensus Meeting

The consensus meeting aimed to reach agreement on the characteristics of a complex intervention aimed at developing clinical leadership of SCNs.

Participants highlighted the importance of a well-structured introductory pathway shared with the organization. This pathway should have clear objectives and ensure ongoing support. The literature confirms this need, emphasizing that organizational support is essential for developing leadership.^12^

It has been suggested that a training package be created that includes key topics such as clinical leadership, communication, effective collaboration, and empowerment. Previous studies show that clinical leadership is not only based on technical skills, but also requires communication skills, reflection on practice, and a sense of responsibility.^35–38^

According to participants, using simulations and comparing experiences are key tools to foster individual and group growth. In addition, it was considered essential to begin with a self-assessment of one’s leadership so as to identify the most relevant content for training.^12^

Leadership development should address both individual and organizational needs, using appropriate training tools and support.

The consensus meeting emphasized the importance of a structured and shared pathway, appropriate training, and active participation of SCNs in decision making to develop their clinical leadership effectively and sustainably. In addition, participants highly valued their involvement in decision-making to identify and plan the development pathway, promoting a sense of empowerment and active participation. This shared decision-making approach between nurses and nurse managers is associated with better patient outcomes and a better work culture.^28–30^,^39^

### Clinical Practice Implication and Next Steps

This study suggests that the development of a structured intervention to develop the clinical leadership of SCNs may have important implications for professionals and healthcare services. In particular, a training pathway that integrates the development of competencies in leadership, communication and collaboration may contribute to strengthening professional trust, work engagement and resilience of nurses, favouring at the same time greater work satisfaction and staff retention. At an organizational level, reinforcing clinical leadership supports improved team coordination, better quality care and patient safety. Organizational support and development of professional competencies are key factors to face challenges relating to sustainability of the nursing workforce and improvement of nursing care outcomes.^34^,^40^,^41^

### Strengths and Limitations

This is one of the few studies where a complex intervention was developed to promote clinical leadership.

The limitations of the study are that it took place in only one hospital setting, so it may not be generalizable; however, the results obtained may be transferable to similar settings.

One limit to this study relates to the limited number of participants, single institution involved, as well as the definition of the role of the SCNs which is strongly influenced by the local organizational context – factors which limit the transferability of the results to other healthcare contexts. However, these results represent useful first steps to identify key components of an intervention aimed to develop clinical leadership of SCNs. In line with the Medical Research Council Framework for the development of complex interventions, future studies should concentrate on the evaluation of feasibility and acceptability of the proposed training pathway, as well as the identification of appropriate outcome indicators such as the development of leadership competencies, working climate and care outcomes.

## Conclusion

In conclusion, the findings of this study demonstrate that SCNs perceive their role and clinical leadership development as a dynamic process rooted in personal skills, interprofessional collaboration, and a supportive organizational culture. Specifically, the analysis identified five macro themes: 1. Personal characteristics and competencies, 2. Collaboration and Relationships, 3. Organization and culture of teams, 4. Leadership in the role and 5 patient. These elements are perceived by SCNs and experts as critical drivers for leadership, requiring constant critical reflection and the ability to lead change.

Within the analyzed context, these findings suggest that enhancing the SCN role could lead to a more positive organizational climate, promoting transparency and accountability. While the literature and the participants’ perceptions—captured in the “patient” macro-theme—point toward a correlation between clinical leadership and continuous improvement in care quality, it is important to clarify that this study did not directly measure patient outcomes. Instead, these should be viewed as intended implications and expected benefits of the identified strategies.

Furthermore, this research identified the components for a complex intervention to support SCNs, structured around personal and professional development through training, coaching, and participatory governance. However, given the qualitative, single-site nature of this research, these findings must be interpreted within their specific context, as their transferability to different healthcare settings may be limited.

As a next step, it is essential to conduct a feasibility and implementation evaluation of the proposed intervention. Future research should incorporate clear performance indicators—such as staff turnover rates, incident reporting frequency, or validated leadership self-assessment tools—to rigorously evaluate the actual impact of SCN leadership on clinical and organizational outcomes.
